# The interaction between personality traits and dysfunctional breathing during the COVID-19 pandemic in Russians

**DOI:** 10.1192/j.eurpsy.2021.824

**Published:** 2021-08-13

**Authors:** J. Koniukhovskaia, E. Pervichko, O. Mitina, O. Stepanova, E. Dorokhov

**Affiliations:** 1 Psychology, Lomonosov Moscow State University, Moscow, Russian Federation; 2 Faculty Of Psychology, Lomonosov Moscow State University, Moscow, Russian Federation; 3 Faculty Of Psychology And Social Sciences, Pirogov Russian National Research Medical University, Moscow, Russian Federation

**Keywords:** personality traits, HEXACO, dysfunctional breathing, COVID-19 pandemic

## Abstract

**Introduction:**

The COVID-19 pandemic situation creates specific conditions for increased anxiety and increased attention to respiratory sensations. This can become a favorable ground for the occurrence of dysfunctional breathing. Dysfunctional breathing is a pattern of breathing that does not meet physiological needs and can lead to respiratory, cardiovascular, digestive disorders and neurological dysfunctions (Chaitow et al.,2014)

**Objectives:**

The aim of the study is to identify “personality predictors” for the occurrence of dysfunctional breathing in the Russian population during the COVID-19 pandemic.

**Methods:**

The author’s socio-demographic questionnaire, the Naimigen Questionnaire (VanDixhoorn, Duivenvoordent, 1984), HEXACO-PI-R (Ashton, Lee, 2017; Egorova, Psrshikova, Mitina, 2019), and The State-Trait Anxiety Inventory (Spielberger, 1983; Leonova, 2013) were used. The study was conducted online from April 27 to May 27. 582 people from all regions of Russia attended it, including 496 women and 86 men aged 18 to 64 years.

**Results:**

Dysfunctional breathing has a direct correlation with personal anxiety (r=0.543, p=0.000) and emotionality (r=0.370,p=0.000), as well as a negative correlation with the personality traits of Honesty/ Humility(r=-0.153, p=0.000), Extraversion (r=-0.247, p=0.000), Agreeableness (r=-0.226, p=0.000), and Conscientiousness (r=-0.128, p=0.002).

**Conclusions:**

Thus, in the COVID-19 pandemic context, dysfunctional breathing was detected in people with increased trait anxiety and pronounced emotionality, as well as in people with hostility and low conscientiousness/organization, as well as in introverts and those who are inclined to demonstrate social status. The occurrence of dysfunctional breathing during a pandemic can be interpreted as a sign of coronavirus disease by those people, which can motivate them to seek medical help, and thus increase the burden on the healthcare system.

